# A Multi‐Omics, Machine Learning‐Aware, Genome‐Wide Metabolic Model of *Bacillus Subtilis* Refines the Gene Expression and Cell Growth Prediction

**DOI:** 10.1002/advs.202408705

**Published:** 2024-09-17

**Authors:** Xinyu Bi, Yang Cheng, Xueqin Lv, Yanfeng Liu, Jianghua Li, Guocheng Du, Jian Chen, Long Liu

**Affiliations:** ^1^ Key Laboratory of Carbohydrate Chemistry and Biotechnology Ministry of Education Jiangnan University Wuxi 214122 China; ^2^ Science Center for Future Foods Ministry of Education Jiangnan University Wuxi 214122 China

**Keywords:** cell growth, comprehensive metabolic network model, gene expression, machine learning, multiomics knowledgebase

## Abstract

Given the extensive heterogeneity and variability, understanding cellular functions and regulatory mechanisms through the analysis of multi‐omics datasets becomes extremely challenging. Here, a comprehensive modeling framework of multi‐omics machine learning and metabolic network models are proposed that covers various cellular biological processes across multiple scales. This model on an extensive normalized compendium of *Bacillus subtilis* is validated, which encompasses gene expression data from environmental perturbations, transcriptional regulation, signal transduction, protein translation, and growth measurements. Comparison with high‐throughput experimental data shows that EM_*i*Bsu1209‐ME, constructed on this basis, can accurately predict the expression of 605 genes and the synthesis of 23 metabolites under different conditions. This study paves the way for the construction of comprehensive biological databases and high‐performance multi‐omics metabolic models to achieve accurate predictive analysis in exploring complex mechanisms of cell genotypes and phenotypes.

## Graphical abstract

A comprehensive modeling framework of multi‐omics machine learning and metabolic network models is proposed to explore gene expression and cell growth in complex environments. The model is verified on the gene expression knowledgebase of *Bacillus subtilis*, and it can accurately predict the expression of 605 genes and the synthesis of 23 metabolites under different conditions.

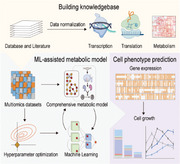

## 1 Introduction

The analysis of multi‐dimensional and highly heterogeneous biological data is one of the main bottlenecks in the current development of biology.^[^ ^1^ ^]^ Multidimensionally, biological systems of interest often involve studies ranging from low‐throughput in situ sequencing analysis to high‐throughput mass spectrometry.^[^ ^2^ ^]^ Regarding heterogeneity, experimental manipulation, sample source, and biological variation can all contribute to observed differences in data from the same or different samples.^[^ ^3^ ^]^ Fortunately, the uncertainty of data analysis provides greater opportunities for the discovery of new questions. After excluding technical and operational errors, inconsistent evaluation of multiple heterogeneous datasets may be the result of biological complexity and nonlinear regulatory mechanisms. In such a case, the discovery of differential data will be an indispensable demonstration for future dissection of complex cellular mechanisms.

With the advancement of high‐throughput omics technology, it has become possible to obtain comprehensive quantitative data on cellular processes. Concomitantly, computational models for analyzing and integrating these datasets have matured. Machine learning (ML) has become a critical tool in biotechnology, with the potential to construct powerful and high‐quality knowledgebases from multi‐omics datasets.^[^ ^4^ ^]^ ML provided a method to identify and predict omics information, which helped reasonably expand the amount of multi‐omics data.^[^ ^5^ ^]^ Besides, ML finds extensive application in building genome scale metabolic network models (GEMs).^[^ ^6^ ^]^ A deep learning‐based computational framework was developed for predicting enzyme commission numbers in GEMs from protein sequences.^[^ ^7^ ^]^ A multimodal ML framework based on fluxomics and transcriptomics was developed to predict cellular phenotypic traits.^[^ ^8^ ^]^ However, many ML models ignored biological context when analyzing data, limiting the credibility of the model and the interpretability of predicted data at the microbial level.^[^ ^9^ ^]^

To improve the analytical accuracy of machine learning models, comprehensive metabolic network models can be combined to systematically capture flux changes in the metabolic network and investigate the impact of gene transcription regulation and protein translation on cellular phenotypes.^[^ ^10^ ^]^ The development of a comprehensive metabolic network model offers an efficient tool for analyzing complex cellular mechanisms in microorganisms. Metabolic and gene expression models covering all components and processes of cellular gene expression have been established, which enables accurate prediction of multi‐scale phenotypes from growth rate to gene expression levels.^[^ ^11^, ^12^ ^]^ The multiscale model has explored physiological mechanisms between gene regulatory networks and metabolic pathways in cellular systems.^[^ ^13^ ^]^

Our aim here was to construct a comprehensive metabolic modeling framework for bacterial organisms that integrates multiscale biological processes and multiple layers of biological organization. We focus on genome‐scale models for *Bacillus subtilis*, a typical model microbial strain commonly used as chassis cells for the production of various enzymes, natural products, functional nutraceuticals, and biomass raw materials.^[^ ^14^ ^]^ GEMs play a crucial role in guiding the design of chassis cells and improving the production of target products in *B. subtilis*. The *i*Bsu1147 model was used to design synthetic pathways for riboflavin and isobutanol.^[^ ^15^ ^]^ The enzyme‐constrained model of ec*i*YO844 was used to engineer strains producing high poly‐γ‐glutamic acid levels.^[^ ^16^ ^]^ The multiscale comprehensive metabolic network model built in our laboratory (et*i*Bsu1209) was used to guide the efficient synthesis of menaquinone‐7(MK‐7).^[^ ^17^ ^]^ A critical aspect of building any high‐accuracy model is to capture and utilize as much experimental data as possible across multiple scales. However, a comprehensive multi‐omics modeling framework that combines models with experimental omics, enabling the incorporation of cell biology knowledge into the learning process, is still lacking.

In this study, we constructed a normalized knowledgebase, including gene expression (496 profiles of 4574 genes), transcriptional regulation (129 transcriptional regulators, 2684 genes), signal transduction (87 transcriptional regulators, 7 environmental stresses), protein translation (4181 arrays) and cell growth (688 arrays). On this basis, we built a comprehensive metabolic network model and conducted a simulation analysis of gene expression and cell growth. To fully combine experimental omics data, we propose a multi‐omics integrated modeling framework that combines 34 machine learning models with a comprehensive metabolic network model to accurately predict gene expression and cell growth based on actual cellular processes. Experimental verification showed that the built model (EM_*i*Bsu1209‐ME) correctly predicted the expression levels of 605 genes with an accuracy of 87.9%, and successfully predicted the synthesis trends of 23 metabolites. This work provides a reference for building a high‐precision comprehensive metabolic network model to explore the complex relationship between cell genotype and phenotype.

## 2 Results

### 2.1 Construction of Gene Expression Knowledgebase

**Figure 1:**
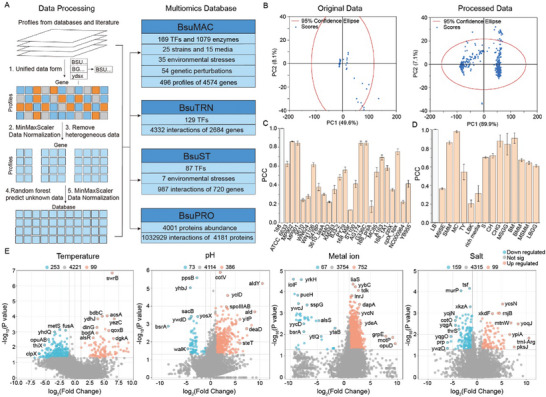
Construction and analysis of multi‐omics knowledgebase of *B. subtilis*. A) Data processing pipeline and multi‐omics data classification of the knowledgebase. B) Principal component analysis of gene expression microarray dataset (BsuMAC) before and after data processing. C) Gene expression diversity was observed among the 25 different *B. subtilis* strains included in BsuMAC. Error bars represent SD. D) Gene expression diversity was observed in *B. subtilis* grown in 15 different media. Error bars represent SD.LB,Luria–Bertani medium; M9SE, M9 Minimal Medium Supplemented with Selenium and Essential Amino Acids; MC, Minimum Culture Medium; SMM, Spizizen minimal medium;TY, Tryptone Yeast Extract Medium; LBK, Luria‐Bertani Medium with Kanamycin; S, Sporulation Medium;CH, Casein Hydrolysate Medium; CHG, Chapman Stone Agar and Glucose medium; MSGG, Minimal Salts Glycerol Glutamate Medium; BM, Belitsky Minimal Medium; BMM, Belitsky Minimal Medium; MSMM, Minimal Salts Mannitol Medium; LBGG, Luria‐Bertani Glycerol Glutamate Medium;. E) Volcano plot of the effects of temperature (22, 30, and 40 °C), pH (6, 7, and 9), metal ions (potassium ions), and salt ions (NaCl) on gene expression in BsuMAC. Unchanged genes are shown in gray, orange indicates upregulated genes, and blue indicates downregulated genes.

Gene expression omics data from multiple databases and extensive literature were collected manually (Table S1, Supporting Information). To standardize various data formats and eliminate any systematic biases originating from experimental conditions and analysis protocols, we established a data normalization strategy for multiple databases, including normalization of multi‐gene IDs by BSU ID, data normalization by MinMaxScaler algorithm, removal of outliers (Orders of magnitude too large or too small), prediction of unknown data by Random Forest, and data normalization by MinMaxScaler algorithm (**Figure** **1**A). The postprocessing data exhibited enhanced diversity, an expanded distribution range, and reduced outliers compared to the original database (Figure 1B). BsuMAC, a gene expression microarray database in *B. subtilis*, was built with 4574 genome‐wide profiles comprising 496 arrays from 15 different culture conditions, 25 strains, 54 genetic perturbations (knockout, overexpression), and 35 environmental stresses (temperature, pH, metal ions, nutrient restriction; Figure 1A and Table S1, Supporting Information). In BsuMAC, environmental stressors exhibit a greater degree of data diversification compared to genetic perturbations (Figure S1, Supporting Information). Among the 25 strains of BsuMAC, the gene expression profiles of *Bacillus subtilis* (*B. subtilis*) strains MP902, MP901, AG174, and G336C showed a strong correlation with that of wild type strain 168, with Pearson's correlation coefficient (PCC) equal to or greater than 0.8 (Figure 1C). Among the 15 media of BsuMAC, the gene expression profiles of Minimum Culture Medium, Spizizen minimal medium, Chapman Stone Agar and Glucose medium, and Belitsky Minimal Medium exhibit robust correlation with Luria–Bertani (LB) medium (PCC≥ 0.8) (Figure 1D). In addition, we also focused on the changes in gene expression profiles in BsuMAC caused by temperature, pH, metal ions, and salts. Metal ions exhibited the most pronounced effect on gene expression, leading to the upregulation of 752 genes and the downregulation of 67 genes (Figure 1E). 253 genes were suppressed under high temperatures, whereas 386 genes were promoted with an increase in pH. However, salt ions only affected the expression of 258 genes.

**Figure 2:**
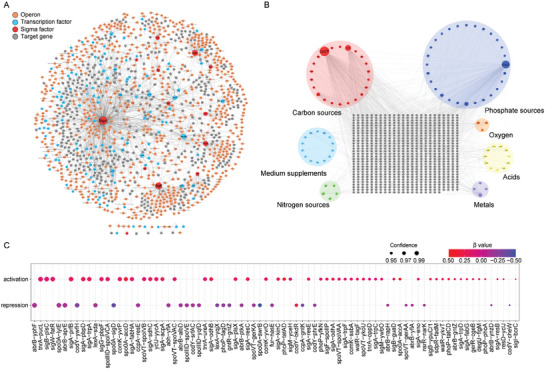
Visualization and confidence analysis of gene regulation datasets. A) Network diagram of all TF‐operon‐targeted genes in BsuTRN. B) Network diagram of signal transduction in *B. subtilis* (BsuST). The gray nodes represent target genes. Nodes represent TFs associated with carbon sources (red), metals (purple), acids (yellow), nitrogen sources (green), oxygen (orange), phosphate sources (dark blue), and medium supplements (light blue). All transcriptional interactions between TFs were represented, including 87 TFs, 7 environmental stressors, 720 target genes, and 987 interactions. C) Confidence and linear regression analyses in BsuTRN. The interaction relationships between 70 TFs and target genes with a confidence level of > 95% are displayed, including 44 activation and 26 repression. The β‐value indicates the intensity of the impact of changes in TFs on changes in target genes.

Visualization of the Transcription Factor (TF)‐operon‐target gene network in BsuTRN showed a comprehensive landscape of gene transcription regulation in *B. subtilis* (**Figure** **2**A; Figure S2, Supporting Information). The network encompassed 129 TFs (including 13 sigma factors), interacting with 742 operons to precisely regulate 857 target genes (Table S2, Supporting Information). The overlap between BsuTRN and BsuST highlighted 87 TF clusters related to seven environmental conditions (Table S3, Supporting Information). The expression of 740 genes was influenced by environmental factors, such as carbon, nitrogen, and phosphate sources, oxygen levels, metal ions, acidity, and culture conditions (Figure 2B). The phosphate and carbon sources exhibited substantial correlations with TFs, involving 28 and 22 TFs, respectively. Confidence and multiple linear regression analyses on the TF‐gene regulatory network were performed to assess the uncertainty of parameter estimates. We found confidence level of the regulatory network involving 80 TF‐genes exceeded 95% (Figure 2C).

### 2.2 Comprehensive Metabolic Network Modeling and Prediction

**Figure 3:**
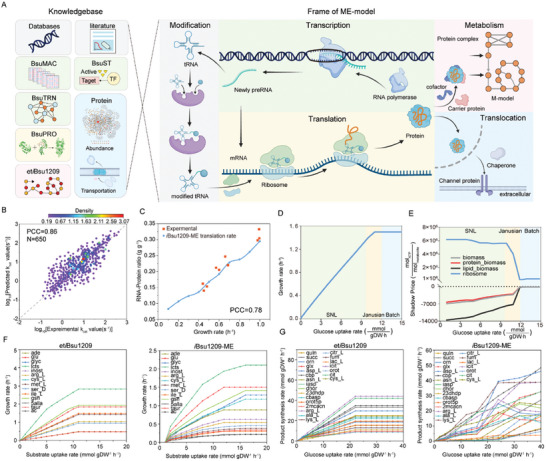
Construction and analysis of *i*Bsu1209‐ME. A) Construction framework of *i*Bsu1209‐ME. Created with BioRender.com. B) Performance verification of the DLKcat deep learning model. The correlation between predicted *k*cat values and *k*cat values from experimental data in *B. subtilis* was evaluated. The brightness of the color represents the density of data points. Student's *t*‐test was used to calculate *p*‐values for Pearson correlations. N represents the number of genes. C) The *i*Bsu1209‐ME predicts RNA‐to‐protein ratios under coupling constraints, one for constant translation and growth rates. Experimental data were from Scott et al.^[^ ^19^ ^]^ D) Growth curve of *i*Bsu1209‐ME. The cell growth rate is plotted as a function of glucose uptake. E) The role of *i*Bsu1209‐ME constraints in the transition from nutrient limitation to bulk growth, as shown by changes in its shadow price. SNL, Strictly Nutrient‐Limited. F) Comparison of cell growth under different substrates in et*i*Bsu1209 and *i*Bsu1209‐ME. ade, Adenine. glu, glucose; glyc, glycerol; lcts, lactose; inost, myo‐Inositol. arg_L, L‐arginine; cys_L, L‐cysteine; met_L, L‐methionine; ser_D, D‐serine; ile_L, L‐isoleucine; gsn, guanosine; 5alla, allantoin; taur, taurine; ac, acetate. Substrate selection from Bi et al.^[^ ^17^ ^]^ G) A simulation comparison of different metabolite synthesis rates in et*i*Bsu1209 and *i*Bsu1209‐ME. quln, pyridine‐2,3‐dicarboxylate; succ, succinate; orn, L‐ornithine; glx, glyoxylate; asp_L, L‐aspartate; cbp, carbamoyl phosphate; asn_L, L‐asparagine; iasp, iminoaspartate; dhor, (S)‐dihydroorotate; 23dhdp, L‐2,3‐dihydrodipicolinate; cbasp, N‐carbamoyl‐L‐aspartate; orot5p, orotidine 5′‐phosphate; 2mcacn, (Z)‐but‐2‐ene‐1,2,3‐tricarboxylate; arg_L, L‐arginine; ala_L, L‐alanine; lys_L, L‐lysine; citr_L, L‐citrulline; fum, Fumarate; lac_L, L‐lactate; icit, isocitrate; orot, orotate; cit, citrate; cys_L, L‐cysteine. Metabolite selection from Bi et al.^[^ ^17^ ^]^

The gene expression network (BSUme) was constructed based on the gene expression knowledgebase of *B. subtilis*, comprising 4253 protein‐coding open reading frames, 208 RNA genes, 576 transcription units, 32 rRNA modifications, 25 tRNA modifications, 1485 protein complexes, 65 modified protein complexes, and 445 translocation proteins (Tables S4,S5, Supporting Information). The *i*Bsu1209‐ME was constructed based on BSUme, including 12032 metabolites, 18115 reactions, and 12089 process data (**Figure** **3**A and **Table** **1**; Table S5, Supporting Information). Given the constraints of limited enzyme data, DLKcat was utilized to extend the *i*Bsu1209‐ME's coverage of enzyme turnover numbers (*k*cat).^[^ ^18^ ^]^ To prove its usability, DLKcat was used to predict 650 *k*cat values in *i*Bsu1209‐ME. The DLKcat's predicted values showed a strong correlation with the experimental values collected from the BRENDA and SABIO‐RK databases (PCC = 0.86) (Figure 3B; Table S5, Supporting Information). On this basis, *k*cat values were predicted for all enzymes in *i*Bsu1209‐ME, resulting in an augmentation of the total *k*cat dataset from 650 to 1847, representing a 2.84‐fold increase (Figure S3, Supporting Information). *i*Bsu1209‐ME encompassed all major metabolic pathways, biomass synthesis pathways, transcription, translation, macromolecular modifications, and translocation reactions. To assess the impact of RNA and protein synthesis rates on cell growth, the model‐predicted RNA‐to‐protein ratio demonstrated a high degree of correlation with experimental data (PCC = 0.78) (Figure 3C).

**Table 1:** Overview of All ProcessData Subclasses in iBsu1209‐ME.

Process Data subclass	Information contained	Example	Number in *i*Bsu1209‐ME
Stoichiometric Data	Metabolite stoichiometry of a metabolic reaction	R05661	1942
Complex Data	Protein subunit stoichiometry of an enzyme complex and the modifications	CPLX8J2‐33	1564
Subreaction Data	Multistep‐related reactions of catalytic enzymes and stoichiometry	Mod_2fe2s_c	187
Transcription Data	Nucleotide sequence, RNA products, sigma factor usage, etc.	Tu_BSU35640	3561
translation Data	tRNA‐mediated amino acid additions, sequence of mRNA/protein, etc.	BSU01020	4103
tRNA Data	Codon, amino acid, tRNA, and modifications required to make a functioning tRNA	BSU_tRNA_83	173
Translocation Data	Keff, enzymes, and metabolite stoichiometry of specific protein translocation pathways	srp	9
Post Translation Data	Translocation pathways, protein modifications (for lipoproteins), etc.	Tran BSU38700	527
Generic Data	List of complexes or metabolites that are redundant and represented as generics	generic_Tuf	13

To explore the performance of *i*Bsu1209‐ME, we verified the cell growth prediction and prediction accuracy of *i*Bsu1209‐ME. The growth simulation process of *i*Bsu1209‐ME resulted in three different growth regions compared with experimental data^[^ ^17^ ^]^: 1) strictly nutrient‐limited (SNL) region, where the growth increases linearly with increasing glucose availability; 2) batch growth region, where the growth rate is not affected by the increase in glucose; and 3) Janusian region, where is situated between the SNL and batch growth regions (Figure 3D). As shown in Figure 3E, *i*Bsu1209‐ME achieved reliable maximum growth rate predictions without limiting nutrient input, which is consistent with empirical models of microbial growth.^[^ ^20^ ^]^ The flux variability of the central carbon metabolites (pyruvate and citrate) in *i*Bsu1209‐ME effectively disappeared increasing the solution accuracy to 10, which is 7 orders of magnitude lower than the minimum flux variability in et*i*Bsu1209 (Figures S4,S5, Supporting Information).

To assess the predictive capability of *i*Bsu1209‐ME, the model was utilized to predict cell growth rates under minimal media conditions with 14 diverse carbon and nitrogen sources (Figure 3F). At substrate uptake rates of 0 to 1 mmol gDW^−1^ h^−1^, the simulated cell growth rate in *i*Bsu1209‐ME is notably constrained, in stark contrast to et*i*Bsu1209.^[^ ^17^ ^]^ The growth rate curves of *i*Bsu1209‐ME exhibited distinct trends with different substrate uptake rates, implying variations in substrate utilization and metabolic pathways (Figure 3F). To delve deeper into disparities in phenotypic predictions, a reanalysis was conducted on the 23 most productive metabolite synthesis pathways utilizing glucose as a substrate reported in the literature.^[^ ^21^ ^]^ The variation in metabolite synthesis carved with escalating glucose uptake rates signifies distinctions in the gene expression patterns of diverse metabolic pathways in *i*Bsu1209‐ME (Figure 3G). Meanwhile, we evaluated the expression levels of 30 genes (central carbon cycle) under 12 diverse culture conditions in *i*Bsu1209‐ME, which had a strong correlation with experimental data (PCC = 0.72) (Figure S6, Supporting Information).^[^ ^22^ ^]^

### 2.3 Comparison of 34 Machine‐Learning Models

**Figure 4:**
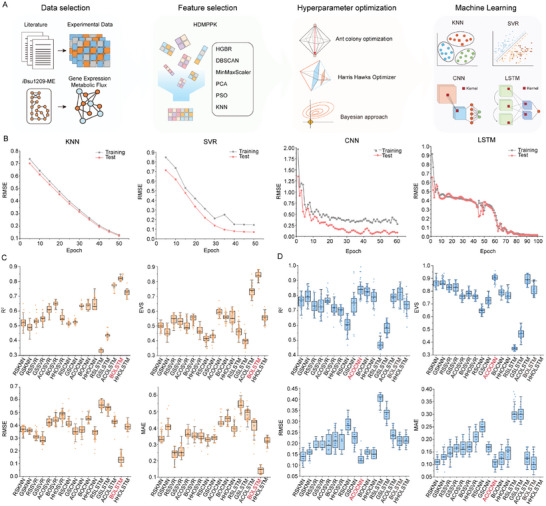
Construction and analysis of multi‐omics Machine learning (ML) models. A) Framework for multi‐omics ML models. HGBR, HistGradientBoostingRegressor. DBSCAN, Density‐Based Spatial Clustering of Applications with Noise. PCA, Principal Component Analysis. PSO, Particle Swarm Optimization. KNN, K‐Nearest Neighbors. SVR, Support Vector Regression. CNN, Convolutional Neural Networks. LSTM, Long Short‐Term Memory. B) The root mean squared error (RMSE) of four ML models (KNN, SVR, CNN, and LSTM) in predicting cell growth during iterative training. C) Comparison of the predictive performance of 17 different ML models on the transcription dataset. D) Comparison of the predictive performance of 17 different ML models on the translation dataset. RSKNN, random search optimized KNN; GSKNN, grid search optimized KNN; RSSVR, random search optimized SVR; GSSVR, grid search optimized SVR; ACOSVR, ant colony optimized SVR; BOSVR, bayesian optimized SVR; HHOSVR, Harris Hawk optimized SVR; RSCNN, random search optimized CNN; GSCNN, grid search optimized CNN; ACOCNN, ant colony optimized CNN; BOCNN, bayesian optimized CNN; HHOCNN, Harris Hawk optimized CNN; RSLSTM, random search optimized LSTM; GSLSTM, grid search optimized LSTM; ACOLSTM, ant colony optimized LSTM; BOLSTM, bayesian optimized LSTM; HHOLSTM, Harris Hawk optimized LSTM.

Mechanistic modeling and flux analysis of comprehensive metabolic network models rely on systematically integrated high‐throughput data.^[^ ^23^ ^]^ In terms of the amount of omics data, gene expression microarray data of *B. subtilis* totaled 509, which was only 18.5% of that of *E. coli* (Figure S7, Supporting Information). The lack of omics data limited the prediction accuracy and application range of *i*Bsu1209‐ME. Therefore, machine learning models were developed to expand gene expression and cell growth datasets required for constructing a comprehensive metabolic network model. These models utilize the above gene expression dataset (592 in 668 arrays) (including protein translation and transcriptional regulation) as input and cell growth rate as output.^[^ ^22^, ^24^, ^25^, ^26^ ^]^ Simulation data (GE‐MF) of the *i*Bsu1209‐ME model (76 in 668 arrays) serves as an extension of the test dataset (**Figure** **4**A). Moreover, the HDMPPK feature engineering scheme [Integrated framework of six algorithms: HistGradientBoostingRegressor (HGBR), Density‐Based Spatial Clustering of Applications with Noise (DBSCAN), MinMaxScaler, Principal Component Analysis (PCA), Particle Swarm Optimization (PSO), and K‐Nearest Neighbors (KNN)] was proposed to improve the quality of the dataset. We optimized the experimental dataset using HDMPPK, and validated the simulation fit with a support vector machine model, contrasting results with the Sparse Group Lasso (SGL) algorithm. The HDMPPK‐processed dataset achieved the highest coefficient of determination (R^2^) at 0.89 and the lowest root mean square error (RMSE) of 0.15 (Figure S8, Supporting Information), which outperformed the SGL method.

To fully compare the impact of machine learning, feature selection, and multi‐view data integration methods on model prediction accuracy, four machine learning models were employed: k‐nearest neighbor (KNN), support vector regression (SVR), convolutional neural network (CNN), and long short‐term memory (LSTM). KNN was chosen for its simplicity and versatility in handling multimodal data.^[^ ^27^ ^]^ SVR was selected for its flexibility in tuning kernel parameters and its effectiveness in managing high‐dimensional datasets.^[^ ^8^ ^]^ CNN demonstrated its capability to capture complex data structures and its suitability for parallel computing.^[^ ^18^ ^]^ LSTM was preferred for predicting multi‐omics data due to its transfer learning capabilities, which facilitate faster task training.^[^ ^28^ ^]^ Hyperparameters govern the architecture and training parameters of the ML model, crucially influencing the performance and generalization capability of the ML model.^[^ ^29^ ^]^ In addition to utilizing traditional grid search and random search algorithms, three state‐of‐the‐art optimization algorithms (ant colony optimization algorithm^[^ ^30^ ^]^, Harris Hawks optimization algorithm^,[^ ^31^ ^]^ and Bayesian optimization algorithm^[^ ^8^ ^]^) were employed to identify the optimal hyperparameter settings with the use of validation data subsets. The ant colony optimization algorithm directs the search process using heuristic pheromones, demonstrating strong robustness in tackling complex combinatorial optimization problems.^[^ ^30^ ^]^ The Harris Hawks optimization algorithm excels in global search capabilities and fast convergence, making it suitable for a wide range of optimization tasks.^[^ ^31^ ^]^ The Bayesian optimization algorithm is effective for global black‐box function optimization and is widely used for hyperparameter tuning.^[^ ^8^ ^]^ The combination of two transcription datasets (experimental data ^[^ ^22^, ^24^ ^]^ and GE‐MF) and two translation datasets (experimental data ^[^ ^25^, ^26^ ^]^ and GE‐MF), along with the utilization of five optimization algorithms and four ML models, resulted in a total of 34 scenarios for ML model development.

The influence of varied hyperparameters on model performance was assessed using learning curves (Figure 4B; Figure S9, Supporting Information). The RMSE of model predictions exhibited a gradual decrease with increasing epochs, where each epoch represents one iteration of the dataset through the model predictions. We repeated the training–test procedure 100 times for each ML model to the mitigate uncertainty and randomness inherent in predictions. The Bayesian‐optimized LSTM (BOLSTM) model achieved the highest R^2^ value of 0.83 and the lowest RMSE of 0.12 In the transcription dataset (Figure 4C). Harris Hawk‐optimized CNN (HHOCNN) and Bayesian‐optimized SVR (BOSVR) models exhibited favorable prediction accuracy, with the highest R^2^ values of 0.64 and 0.67, respectively. The predictive outcomes indicated that grid search and random search algorithms are constrained in finding optimal solutions for CNN and LSTM models due to limitations in search space. The ant colony‐optimized CNN (ACOCNN) model demonstrated the most optimal predictive performance in the translation dataset (R^2^ = 0.85 and RMSE = 0.14) (Figure 4D). Furthermore, BOLSTM and ant colony‐optimized SVR (ACOSVR) models attained R^2^ values of 0.81 and 0.76, respectively. Compared with machine learning, the deep learning model (BOLSTM) embodies the ability to effectively capture long‐term dependencies and learn adaptively.

### 2.4 Contribution of HDMPPK to Functional Analysis in the ML Model

**Figure 5:**
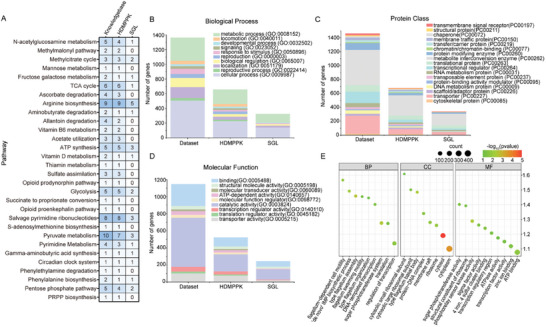
Functional analysis of datasets extracted by feature engineering. A) Characteristic classification of metabolic pathways corresponding to genes selected by dataset, HDMPPK, and SGL. Functional classification of genes selected from dataset, HDMPPK, and SGL based on GO biological processes B), protein class C), and molecular function D). E) GO enrichment analysis of the dataset extracted through HDMPPK.

Gene Ontology (GO) enrichment analysis was performed on the datasets in the ML model to unravel the biological significance. The HDMPPK‐processed databases include all 30 metabolic pathways from the original knowledge base, whereas SGL only contains 16 (**Figure** **5**A). The HDMPPK‐processed databases contain all genes related to important metabolic pathways such as Adenosine Triphosphate synthesis, glycolysis, and tricarboxylic acid cycle (Figure 5A). In addition, we analyzed the genes related to cell growth in the database from three aspects: biological processes, cellular components, and molecular functions. In the analysis of biological processes, the HDMPPK‐processed databases contain protein localization and transcriptional regulation involving transcription factors and translation initiation and elongation factors (Figure 5B,E). In the analysis of protein components, the HDMPPK‐processed dataset contains 17 different enzymes, mainly involved in protein transport, regulation, and modification, which determine the growth rate prediction accuracy in model prediction (Figure 5C,E). In the analysis of molecular functions, catalytic activity, and protein binding are the main data types in the HDMPPK‐processed dataset. In addition to the regulation of gene transcription and translation, the HDMPPK‐processed dataset also encompasses membrane transport proteins and chromosome‐binding proteins. This indicates that the predictions of the ML model take into account the fundamental structure and function of the cell (Figures 5D,E; Figure S10, Supporting Information). These results revealed that the HDMPPK method completely extracted the features of the original database, especially in metabolic and cellular processes.

### 2.5 Optimization of iBsu1209‐ME Through Ensemble Model

**Figure 6:**
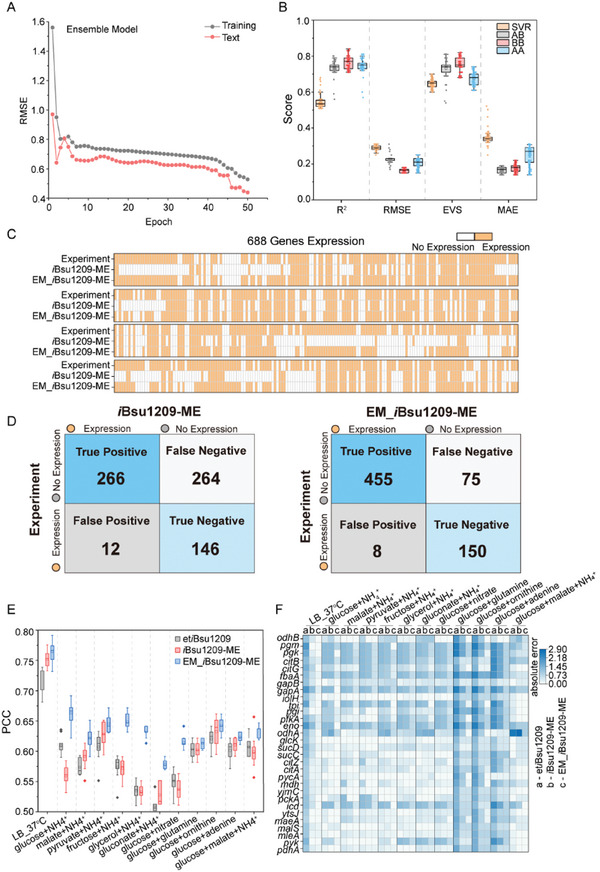
Construction of the ensemble model and optimization of *i*Bsu1209‐ME. A) The root mean squared error (RMSE) of the ensemble model (AB) in predicting cell growth during iterative training. B) Comparison of cell growth prediction performance of four different integrated models. SVR, Support vector regression model for combined transcription and translation datasets; AB, ensemble model of BOLSTM for transcription dataset and ACOCNN for translation dataset; BB, ensemble model of BOLSTM for transcription and translation dataset; AA, ensemble model of ACOCNN for transcription and translation dataset. C) Heatmap of gene expression predictions for *i*Bsu1209‐ME and EM_*i*Bsu1209‐ME. In an LB medium at 37 °C, gene expression predictions for 688 genes in the model were compared to experimental data.^[^ ^22^ ^]^ D) Statistical plot comparing predicted and experimental gene expression data in *i*Bsu1209‐ME and EM_*i*Bsu1209‐ME. E) Comparison of Pearson's correlation coefficient between predicted cell growth values of three models (et*i*Bsu1209, *i*Bsu1209‐ME, and EM_*i*Bsu1209‐ME) and experimental data under 12 different culture conditions. F) Comparison of the absolute errors of the predicted 30 gene expression values by three models (et*i*Bsu1209, *i*Bsu1209‐ME, and EM_*i*Bsu1209‐ME) under 12 different culture conditions with experimental data.^[^ ^22^ ^]^

To comprehensively understand the impact of gene expression on cell growth, we constructed three ensemble models using regression model: AB ensemble models (ACOCNN for translation dataset and BOLSTM for transcription dataset), BB ensemble model (BOLSTM for both translation dataset and transcription dataset), and AA ensemble model (ACOCNN for both translation dataset and transcription dataset). In addition, the SVR model for the translation dataset and transcription dataset was used to predict cell growth, facilitating a comparative analysis of the above three models. The RMSE value of the learning curve gradually decreases as the number of iterations increases by training the above four ensemble models (**Figure** **6**A). The above four ensemble models were simulated and repeated 100 times to remove uncertainty in model predictions (Figure 6B). Unexpectedly, the optimal prediction data were demonstrated in the BB ensemble model (R^2^ = 0.79 and RMSE = 0.16).

The BB ensemble model was used to predict cell growth under different gene expression conditions in BsuMAC, providing reliable cell growth rates for 496 gene profiles (Table S1, Supporting Information). Based on the reconstructed BsuMAC dataset, EM_*i*Bsu1209‐ME was reconstructed by optimizing the transcription and translation modules in *i*Bsu1209‐ME (File S1, Supporting Information). To explore the relationship between cell growth and gene expression, we selected 688 growth‐related genes for simulation. We then compared the simulation results with the data values reported in the literature (Supporting Information).^[^ ^22^ ^]^ EM_*i*Bsu1209‐ME has an accuracy of 87.9% in predicting gene expression levels (605 out of 688 in the model), which is 46.7% higher than that predicted by *i*Bsu1209‐ME (Figure 6C,D). In addition, cell growth rates were predicted under 12 different culture conditions in et*i*Bsu1209,^[^ ^17^ ^]^ *i*Bsu1209‐ME, and EM_*i*Bsu1209‐ME. The predicted values of EM_*i*Bsu1209‐ME and experimental data showed a strong correlation with a PCC of 0.77 in LB medium at 37 °C, (Figure 6E). The expression levels of 30 genes under 12 different culture conditions were predicted in the above three models (Figure 6F). The predicted values of EM_*i*Bsu1209‐ME exhibited an absolute error approaching zero, particularly in forecasting the expression of genes involved with the central carbon cycle, such as *pgk*, *tpi*, and *pgi*.

## 3 Discussion

The construction of machine learning models and comprehensive metabolic network models are inseparable from the support of massive data.^[^ ^8^, ^11^ ^]^ However, there are huge differences in data types and orders of magnitude in databases due to different experimental conditions. To this end, we developed a data processing solution for data normalization, removal of outliers, and data integration based on ML algorithms. We built a multi‐omics knowledgebase covering gene expression, signal transduction, and protein abundance in *B. subtilis*. This multi‐omics knowledgebase provided convenience for training ML, building a comprehensive metabolic network model, and analyzing multi‐omics data to mine potential interactions. Notably, this *B. subtilis* multi‐omics knowledgebase enabled us to analyze genome‐wide molecular expression from signatures of environmental stresses and genetic perturbations. Compared to the previous transcriptional regulation database^[^ ^24^, ^32^ ^]^ and protein expression database^[^ ^26^, ^33^, ^34^ ^]^ in *B. subtilis*, this multi‐omics knowledgebase focused more on linkages between gene expression rather than individual physiological mechanisms of each molecule.

Comprehensive metabolic network models are essential tools for analyzing cell growth metabolic processes in microorganisms^[^ ^11^, ^12^ ^]^. This work constructed a comprehensive metabolic network model in *B. subtilis* (*i*Bsu1209‐ME). In addition to collecting data on gene expression, the *i*Bsu1209‐ME model also defines physiological mechanisms during cell growth, such as ribosomal subreactions, rRNA modifications, and RNA polymerase degradation mechanisms. Most importantly, *i*Bsu1209‐ME enables the correlation analysis and prediction of gene expression and metabolic flux. In the same way as the comprehensive metabolic network models of *E. coli* ^[^ ^11^ ^]^ and *S. cerevisiae*,^[^ ^13^ ^]^ the establishment of *i*Bsu1209‐ME provides model support for physiological research and directed evolution of gene expression in *B. subtilis*.

A challenge in developing comprehensive metabolic network models is obtaining high‐quality omics data to enhance prediction accuracy^[^ ^1^ ^]^. This study develops a machine learning framework that expands the gene expression‐cell growth dataset. The most significant work was the establishment of a comprehensive and detailed ML framework that included dataset selection, feature engineering strategies, hyperparameter optimization, and ML algorithm comparisons. We proposed a new feature engineering framework called HDMPPK, which aims to solve challenges brought about by complex data types and unordered feature extraction. We systematically investigated the disparities generated by diverse hyperparameter optimization algorithms at each ML model and identified the optimal combination to elucidate the relationship between gene expression and cell growth rate. Notably, the simulation data of the comprehensive metabolic network model expands the data set for the machine learning model, and the machine learning model provides optimized data for the comprehensive metabolic network model. This virtuous cycle based on experimental data provides a large amount of high‐quality data for model construction and optimization. The establishment of this ML framework provided a technical approach for diversity mining of omics data and exploring complex genotype‐phenotype relationships.

In conclusion, this study developed a multi‐omics integrated framework combining machine learning and a comprehensive metabolic network model to expand the gene expression‐cell growth dataset. We established a multi‐omics knowledgebase for *B. subtilis* and provided data support for the construction of comprehensive metabolic network models and machine learning models. We constructed EM_*i*Bsu1209‐ME based on the integrated ML model, which effectively improved the prediction accuracy of the model at the gene expression level. These works provide valuable data support for comprehensively exploring genotype‐phenotype relationships in *B. subtilis*, guiding directed cell evolution, and revealing unknown cellular processes. Importantly, this work provides technical solutions for data mining and model development of other microorganisms.

## 4 Experimental Section

### Overview of Knowledge Construction

A gene expression profile (BsuMAC) of 4574 genes comprising 496 arrays was constructed by integrating microarray data from GEO databases^[^ ^35^ ^]^ and literature^[^ ^24^, ^32^ ^]^ (File S1, Supporting Information). 169 TFs and 1079 enzymes were identified from the genome using BsuBCyc^[^ ^36^ ^]^, DBTBS^[^ ^37^ ^]^, and SubtiWiki^[^ ^38^ ^]^ (Table S1, Supporting Information). BsuTRN was a transcriptional regulatory network compendium collected from databases and literature,^[^ ^26^, ^33^, ^34^ ^]^ containing 4332 interactions of 127 TFs regulating 2684 genes (Table S2, Supporting Information). BsuST included signal transduction pathways of cells to environmental stimuli;^[^ ^24^, ^25^, ^32^, ^33^, ^39^ ^]^ 987 signal transduction networks of 44 TFs to 720 genes were determined (Table S3, Supporting Information). BsuPro was a protein abundance compendium containing proteome data of 4001 genes obtained through UniProt^[^ ^40^ ^]^ (Table S4, Supporting Information).

### Modeling and Simulation of Comprehensive Metabolic Network Model

The COBRAme framework of *B. subtilis* was comprehensively reconstructed based on the above‐mentioned knowledgebase, which included a ribosome module, transcription module, translocation module, and tRNA charging module.^[^ ^41^ ^]^ Based on this, BSUme was integrated with the previously built et*i*Bsu1209^[^ ^17^ ^]^ to construct the comprehensive metabolic network model (*i*Bsu1209‐ME). Detailed information on the comprehensive metabolic network model building was in BSUme (File S1; Table S5, Supporting Information). Flux balance analysis was used to simulate all experimental conditions and validate the model.^[^ ^42^ ^]^ To simulate growth under different culture conditions, model inputs were modified based on previous studies (Supporting Information).^[^ ^22^ ^]^ Pearson's correlation coefficient was used as a metric to evaluate the correlation between model simulation values and experimental data. The solveME Python module was used to solve the comprehensive metabolic network model using the 128‐bit (quad precision) linear program and the nonlinear program solver Quad MINOS version 5.6 (qMINOS).^[^ ^43^ ^]^

### Machine Learning Models

Scikit‐learn version 0.24.1 functions sklearn.svm.SVR and sklearn. neighbors.KNeighborsRegressor was used to implement SVR and K‐neighbor regressor, respectively. Keras version 2.12.0 was used to realize the construction of CNN and LSTM, respectively (File S1, Supporting Information).

### Hyperparameter Optimization

To obtain the optimal hyperparameters of the model, five optimization algorithms (grid search, random search, ant colony optimization, Harris Hawk optimization, and Bayesian optimization) were explored. The Python Scikit‐learn library (version 0.24.1) was used for the optimization of hyperparameters (File S1, Supporting Information).

### Feature Engineering

The experimental data reported in the literature (592 in 668 arrays) and the simulation data of the *i*Bsu1209‐ME model (76 in 668 arrays) were used as the dataset for machine learning^[^ ^22^, ^24^, ^25^, ^26^ ^]^ (Table S6, Supporting Information). Transcriptomic and proteomic were selected as feature samples. To evaluate model generalization, the samples were randomly split into training and testing subsets comprising 80% and 20% of the main dataset, respectively. To improve the predictive performance and interpretability of the model, a feature engineering scheme called HDMPPK [Integrated framework of six algorithms: HistGradientBoostingRegressor (HGBR), Density‐Based Spatial Clustering of Applications with Noise (DBSCAN), MinMaxScaler, Principal Component Analysis (PCA), Particle Swarm Optimization (PSO), and K‐Nearest Neighbors (KNN)] was proposed. The Sparse Group Lasso (SGL) algorithm was also used for the feature selection of the dataset to compare to the HDMPPK^[^ ^8^ ^]^ (File S1, Supporting Information).

### Performance Metrics in Machine Learning

To evaluate the predictive accuracy and generalization ability of the model, the following four indicators were used:

Coefficient of Determination (R^2^):

(1) $$R^{2}\;=\;1-\frac{\sum_{i=1}^{n}{\left(y_{\mathrm{ture},i}-y_{\mathrm{pred},i}\right)}^{2}}{\sum_{i=1}^{n}{\left(y_{\mathrm{ture},i}-\overset{-}{y_{\mathrm{ture}}}\right)}^{2}}$$

Root Mean Square Error (RMSE):

(2) $$\mathrm{RMSE}\;=\;\sqrt{\frac{1}{n}\sum_{i=1}^{n}{\left(y_{\mathrm{ture},i}-y_{\mathrm{pred},i}\right)}^{2}}$$

Explained variance score (EVS):

(7) $$\mathrm{EVS}=1-\frac{\mathrm{var}\left(y_{\mathrm{ture},i}-y_{\mathrm{pred},i}\right)}{\mathrm{var}\left(y_{\mathrm{ture},i}\right)}$$

Mean absolute error (MAE):

(3) $$MAE\;=\;\frac{1}{n}\sum_{i\;=\;1}^{n}\left|y_{ture,i}-y_{pred,i}\right|$$

where *y*_ture_ is the predicted value of the model, *y*_pred_ is the experimental value, *i* is the index of the data, and *n* is the number of samples.

### Biological Feature Classification

GO enrichment analysis of the dataset used DAVID (david.ncifcrf.gov/conversion.jsp). The biological classification of genes was obtained through the PANTHER classification system.^[^ ^44^ ^]^

## Conflict of Interest

The authors declare no conflict of interest.

## Author Contributions

X.Y.B., J.C., and L.L. proposed and designed all experiments. X.Y.B. and Y.C. built and analyzed the model. X.Y.B., X.Q.L, and Y.F.L. collected and analyzed data. J.H.L., G.C.D., and L.L. suggested the manuscript. Unless otherwise stated, all authors contributed to the writing, review, and editing of the manuscript.

## Supporting information



Supporting Information

Supplemental Table 1

Supplemental Table 2

Supplemental Table 3

Supplemental Table 4

Supplemental Table 5

Supplemental Table 6

## Data Availability

The data that support the findings of this study are available from the corresponding author upon reasonable request.
