# Supplementary material for: A Multi‐Omics, Machine Learning‐Aware, Genome‐Wide Metabolic Model of Bacillus Subtilis Refines the Gene Expression and Cell Growth Prediction
Source: Adv Sci (Weinh). 2024 Sep 17;11(42):2408705. doi: 10.1002/advs.202408705 (PMC11558093; doi:10.1002/advs.202408705)
Supplement: Supplementary file 1 — Supporting Information [file ADVS-11-2408705-s003.docx]

**Supplementary Information**

**A multi-omics, machine learnning-aware, genome-wide metabolic model of *Bacillus subtilis* refines the gene expression and cell growth prediction**

- **Supplementary Figures 1-10.**
- **Supplementary Methods.**
- **Supplementary References.**

## Supplementary Figure:


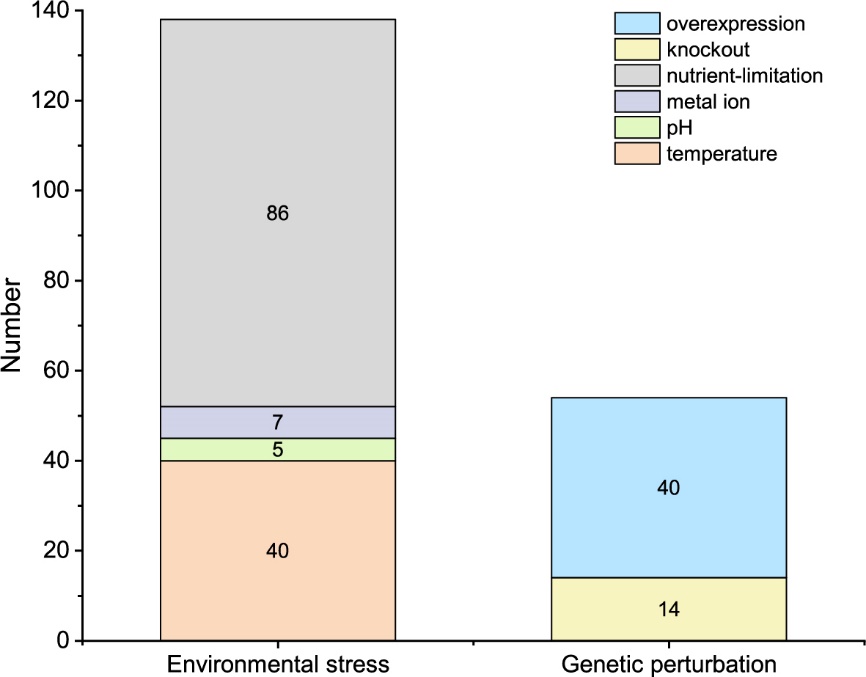


Supplementary Figure 1. Comparison of the number of microarrays in the two categories of environmental stress and genetic interference in the BsuMAC dataset. Environmental pressures mainly include four categories: temperature, pH, metal ions, and nutritional limitations. Genetic interference is mainly divided into gene knockout and overexpression.


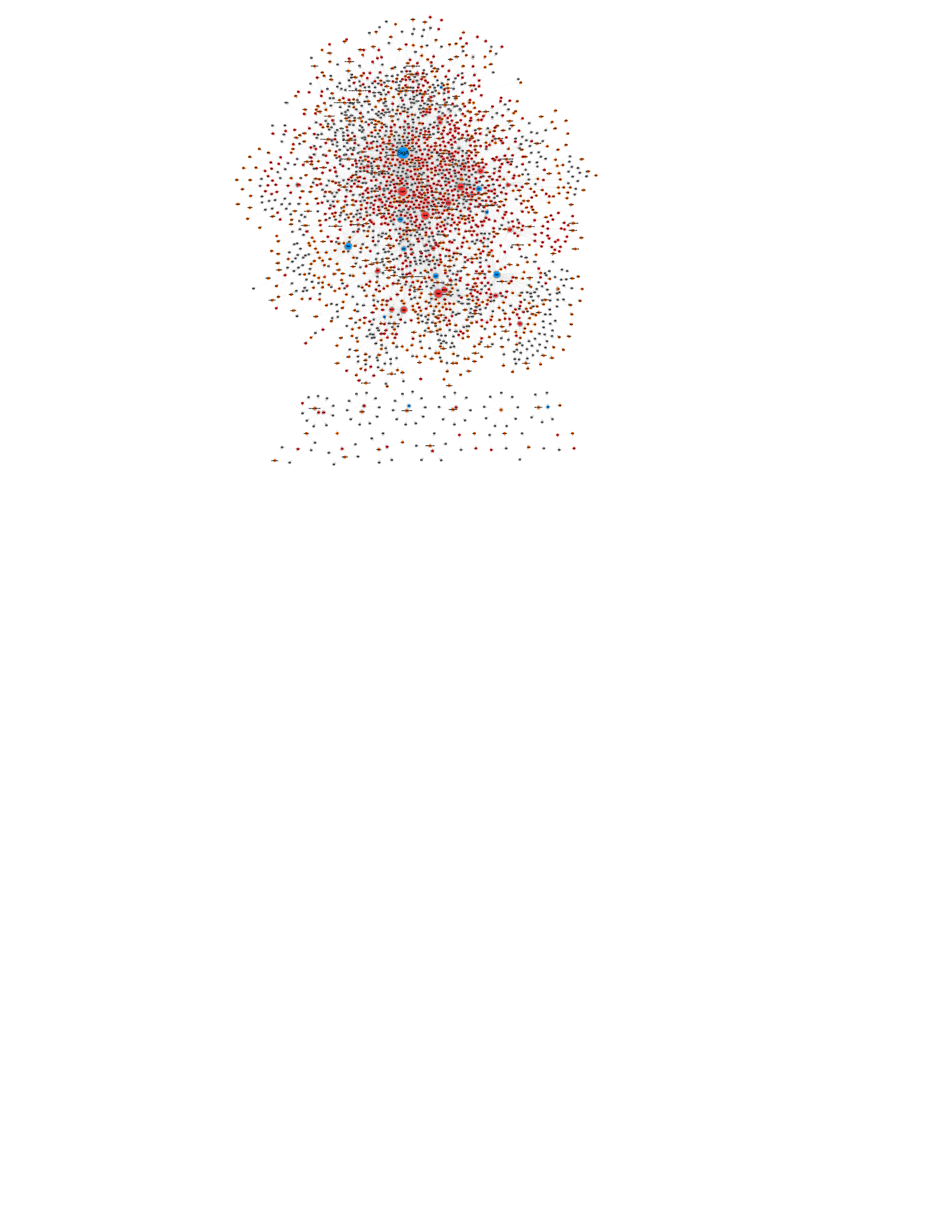


Supplementary Figure 2. Visual network diagram of transcription factors and target genes in the BsuTRN dataset. Blue represents sigma factors, Red represents transcription factors, orange represents operons, and gray represents target genes.


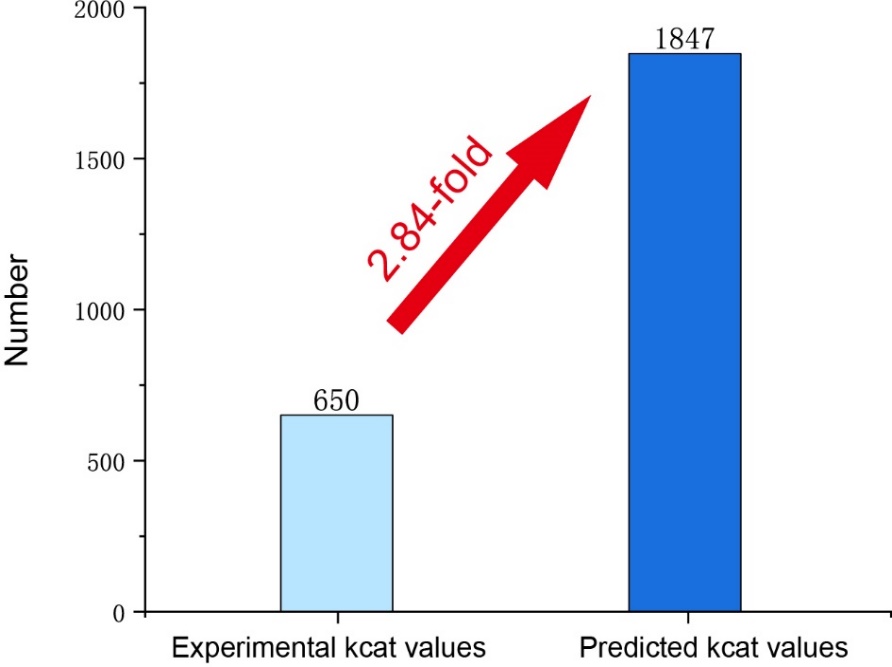


Supplementary Figure 3. Enzyme turnover number comparison between experimental data and predicted data by DLKcat. ^[1]^


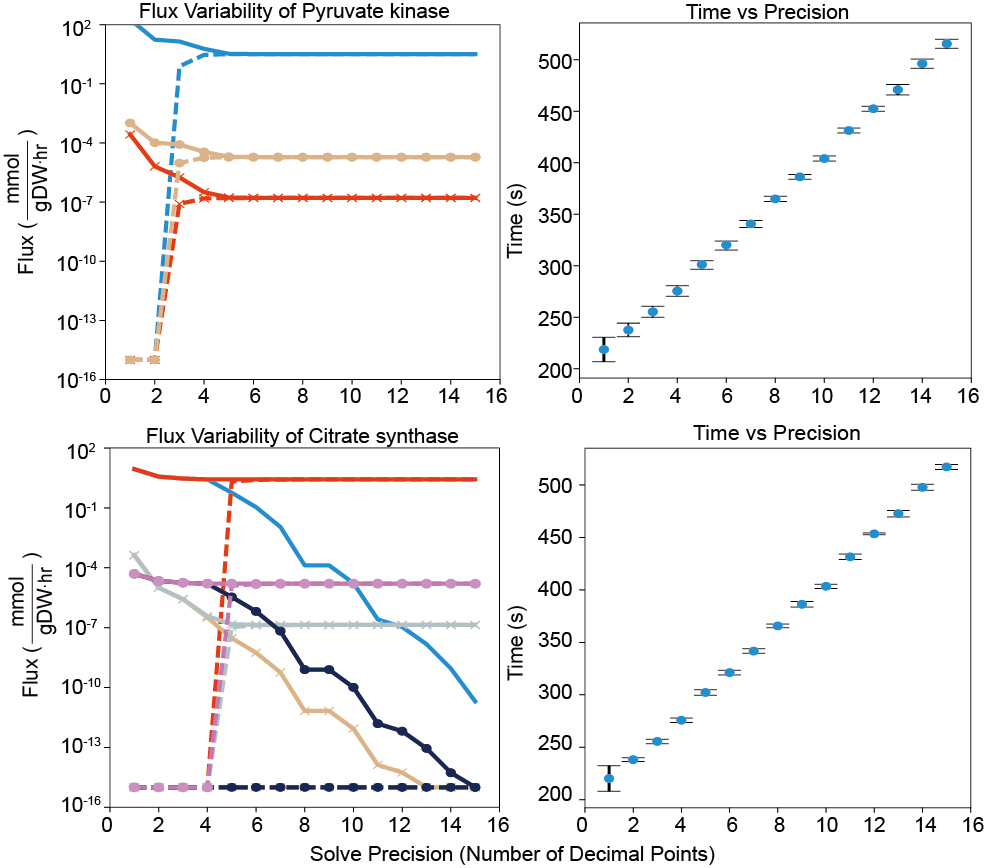


Supplementary Figure 4. Effect of solver accuracy on flux changes for pyruvate and citrate reactions and the reactions required by their synthesizing enzymes in *i*Bsu1210-ME. In the pyruvate flux variation diagram, the blue line represents the metabolic reaction, the red line represents the transcription reaction, and the yellow line represents the translation reaction. In the citrate flux variability plot, red and blue lines represent metabolic reactions, pink and black lines represent transcriptional reactions, and gray and yellow lines represent translational reactions.


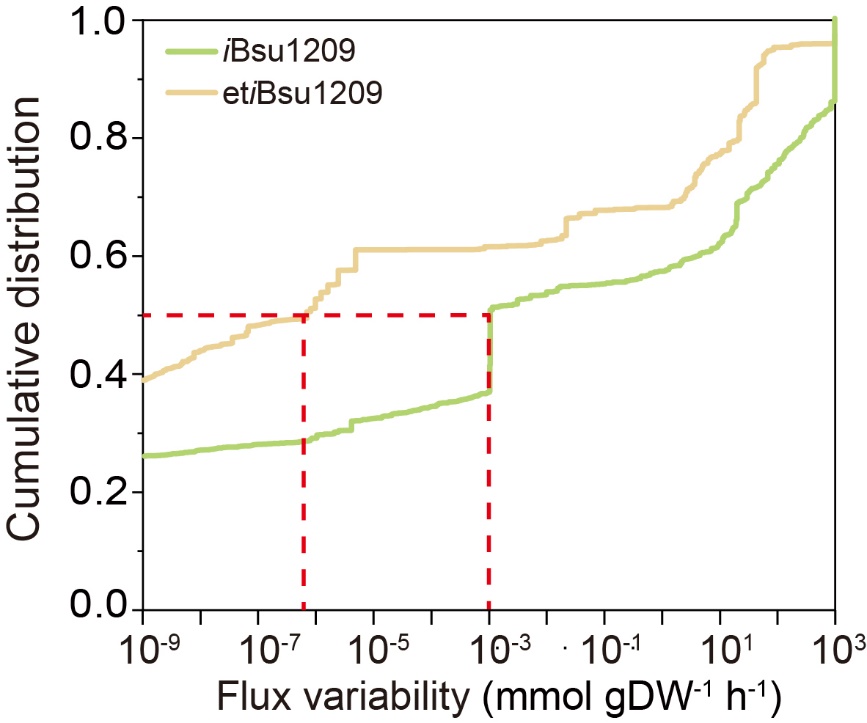


Supplementary Figure 5. Flux variability analysis of built models *i*Bsu1209 and et*i*Bsu1209. ^[2]^


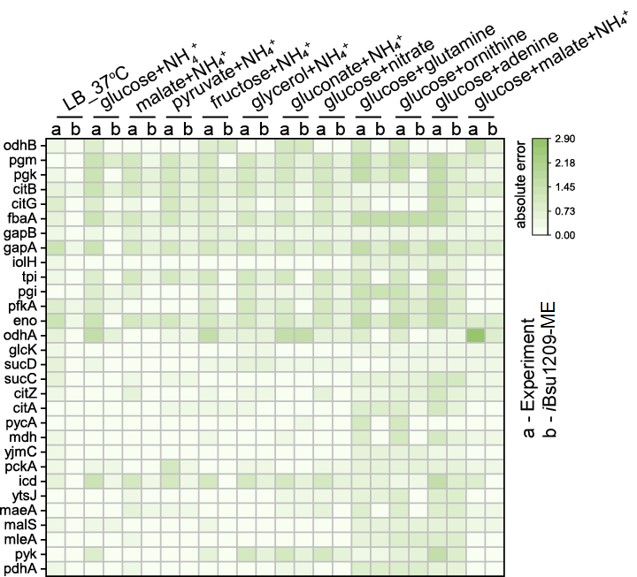


Supplementary Figure 6. The expression levels of 30 genes under 12 different culture conditions were predicted by *i*Bsu1209-ME and experimental data. odhB, 2-oxoglutarate dehydrogenase complex (BSU19360). pgm, phosphoglycerate mutase (BSU33910). pgk, phosphoglycerate kinase (BSU33930). citB, aconitase, trigger enzyme (BSU18000). citG, fumarase (BSU33040). fbaA, fructose 1,6-bisphosphate aldolase (BSU37120). gapB, glyceraldehyde-3-phosphate dehydrogenase (BSU29020). gapA, glyceraldehyde 3-phosphate dehydrogenase (BSU33940). iolH, myo-inositol catabolism (BSU39690). tpi, triose phosphate isomerase (BSU33920). pgi, glucose 6-phosphate isomerase (BSU31350). pfkA, phosphofructokinase (BSU29190). eno, enolase (BSU33900). odhA, 2-oxoglutarate dehydrogenase (BSU19370). glcK, glucose kinase (BSU24850). sucD, succinyl-CoA synthetase (BSU16100). sucC, succinyl-CoA synthetase (BSU16090). citZ, citrate synthase (BSU29140). citA, minor citrate synthase (BSU09440). pycA, pyruvate carboxylase (BSU14860). mdh, malate dehydrogenase (BSU29120). yjmC, ureidoglycolate dehydrogenase (BSU12320). pckA, phosphoenolpyruvate carboxykinase (BSU30560). icd, isocitrate dehydrogenase (BSU29130). ytsJ, bifunctional malic/malolactic enzyme (BSU29220). maeA, malic enzyme (BSU37050). malS, malate dehydrogenase (BSU29880). mleA, malic enzyme (BSU23550). pyk, pyruvate kinase (BSU29180). pdhA, pyruvate dehydrogenase (BSU14580). The experimental data came from literature reports.^[3]^


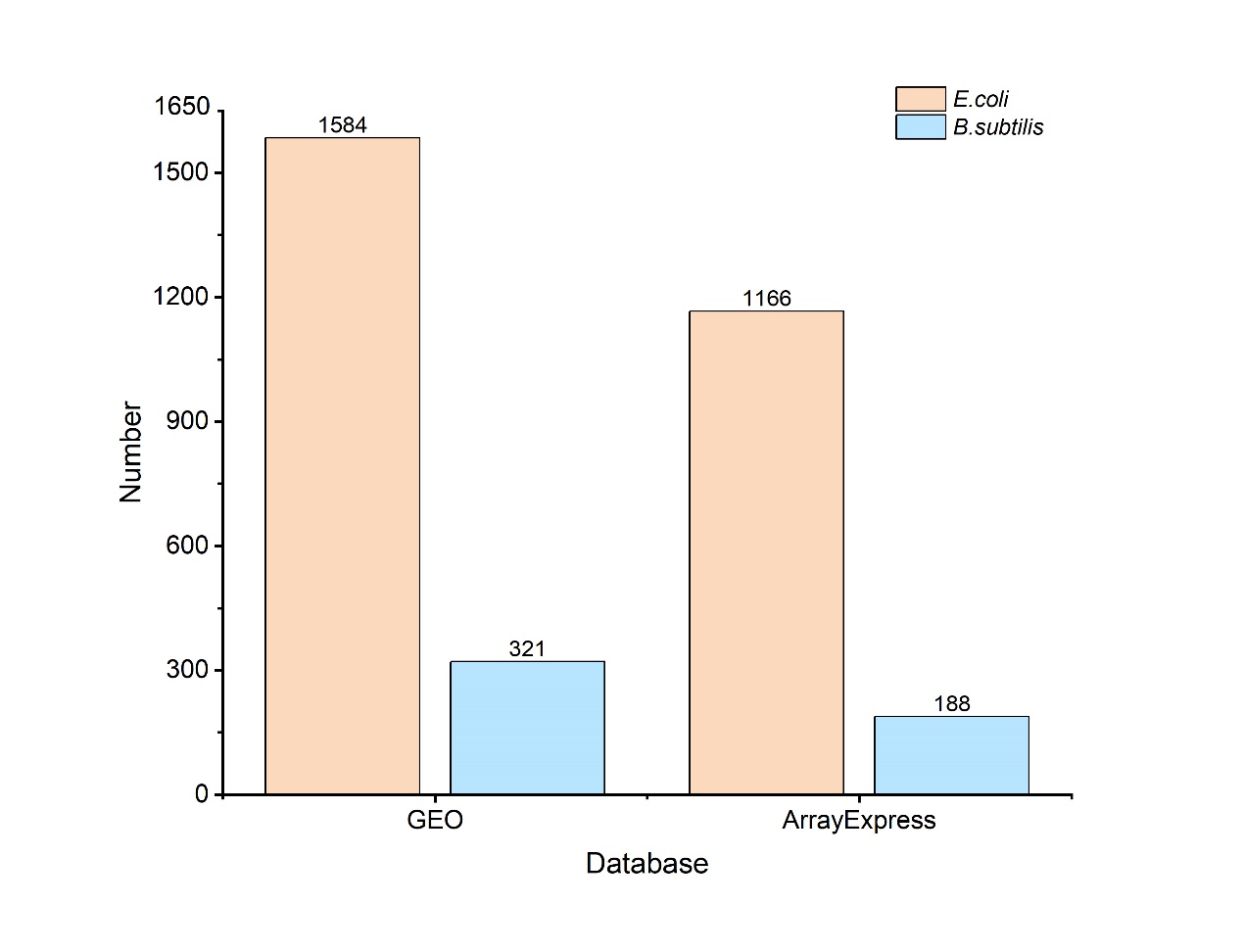


Supplementary Figure 7. Comparison of microarray data volume of *Bacillus subtilis* and *Escherichia coli* in GEO and ArrayExpress databases (including microarray data and RNA-Seq data).


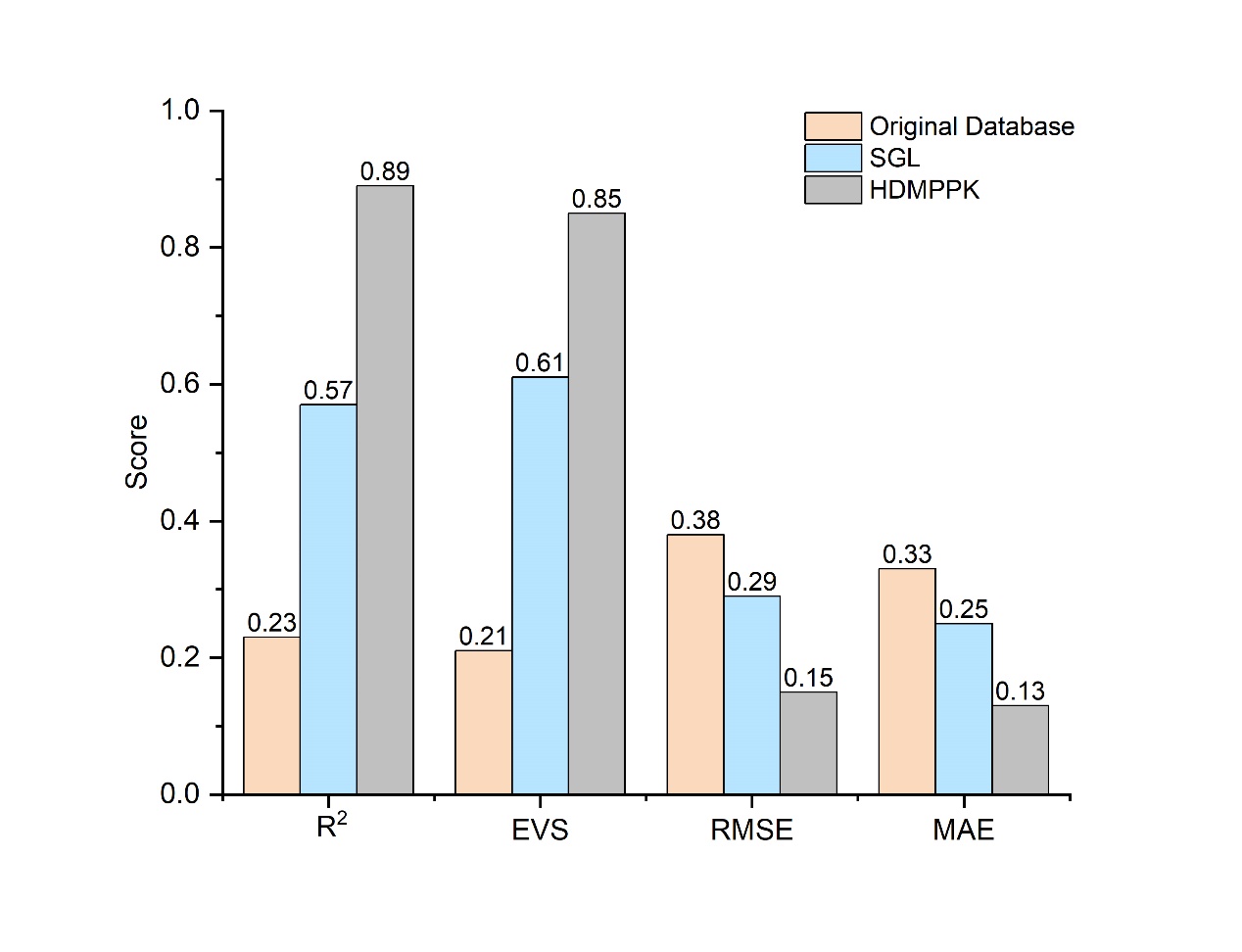


Supplementary Figure 8. Dataset optimization for machine learning via HDMPPK and Sparse Group Lasso (SGL) algorithms.


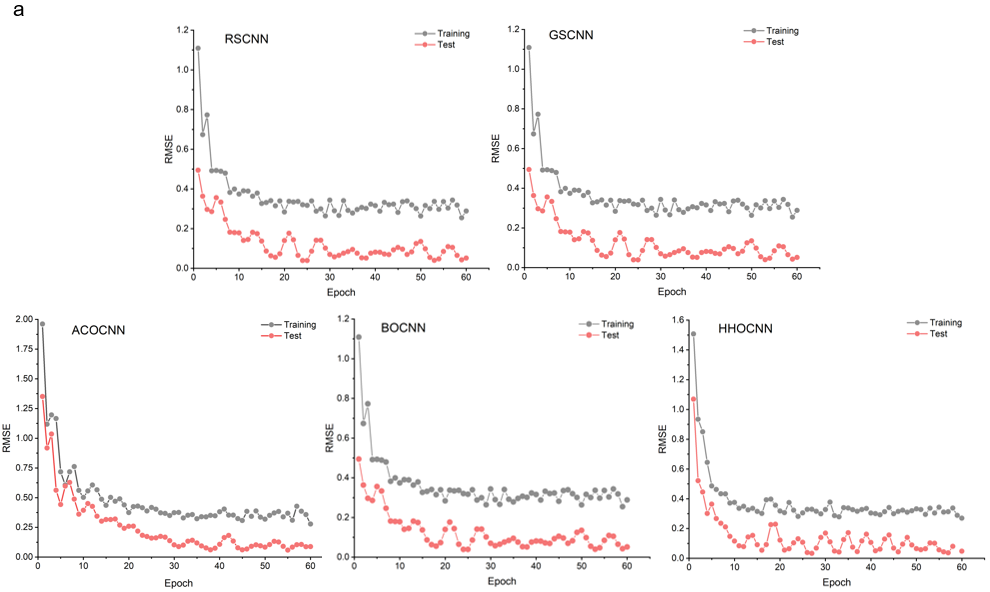


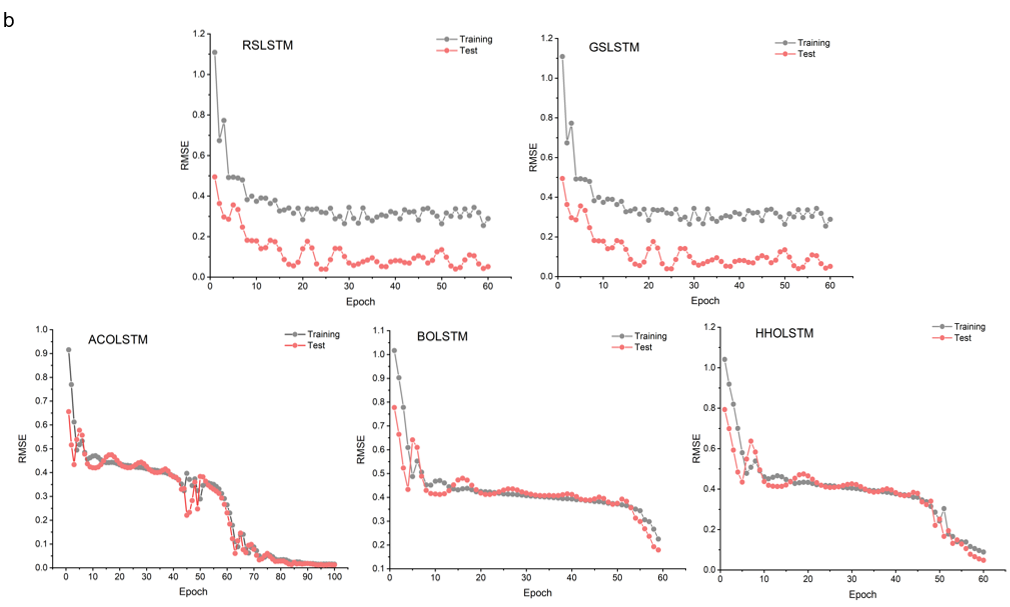


Supplementary Figure 9. Root mean square error (RMSE) of convolutional neural network (CNN) (a) and Long Short-Term Memory (LSTM) (b) in predicting cell growth during iterative training under different optimization algorithms. There is no significant change in the root mean square error (RMSE) curves of KNN and SVR models predicting cell growth during iterative training under different optimization algorithms. RSCNN, Random search Optimized CNN. GSCNN, Grid search Optimized CNN. ACOCNN, Ant Colony Optimized CNN.BOCNN, Bayesian Optimized CNN. HHOCNN, Harris Hawk Optimized CNN. RSLSTM, Random search Optimized LSTM. GSLSTM, Grid search Optimized LSTM. ACOLSTM, Ant Colony Optimized LSTM. BOLSTM, Bayesian Optimized LSTM. HHOLSTM, Harris Hawk Optimized LSTM.


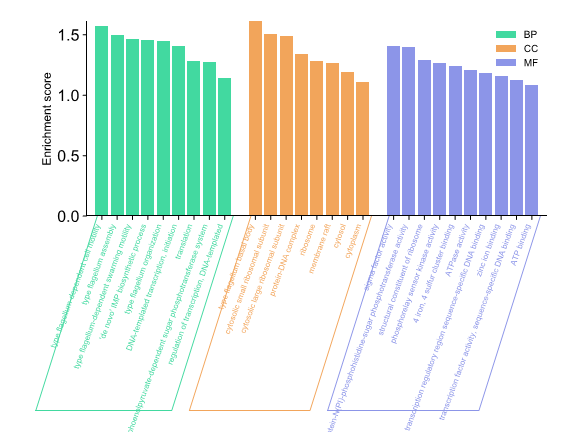

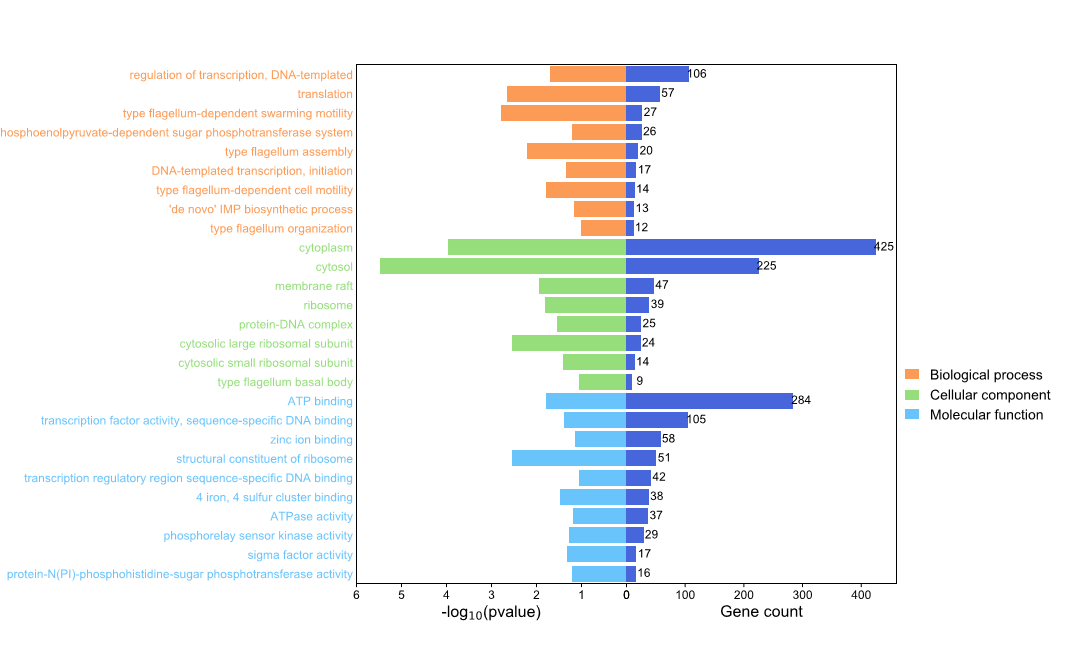


Supplementary Figure 10. Gene ontology enrichment analysis plot for genes from the dataset using HDMPPK. Analyze gene data for Biological Process (BP), Cellular Component (CC), and Molecular Function (MF) in three parts. The above panel assesses the enrichment levels of genes in BP, CC, and MF. The below panel analyzes the significance and gene composition within BP, CC, and MF.

**Supplementary Methods：**

## 1. Machine-Learning Models

Scikit-learn v 0.24.1 functions sklearn.svm.SVR and sklearn.neighbors.KNeighborsRegressor were used to implement support vector regression (SVR) and K-neighbors regressor (KNR), respectively. Keras v2.12.0 was used to realize the construction of a convolutional neural network (CNN) and Long Short-Term Memory Network (LSTM), respectively. The CNN architecture employed in this study is a modified version of the VGG-16 network. It consists of 13 convolutional layers, 5 max-pooling layers, and 3 fully connected layers. Rectified Linear Unit (ReLU) activation functions were applied after each convolutional and fully connected layer. Dropout layers with a rate of 0.5 were inserted after the third and fourth max-pooling layers to prevent overfitting. The model was optimized using the Adam optimizer with an initial learning rate of 0.001. The categorical cross-entropy loss function was utilized for multi-class classification. A learning rate decay schedule with a decay factor of 0.1 was employed every 10 epochs. The batch size was set to 32, and the model was trained for a total of 50 epochs. The LSTM architecture utilized in this study consists of two LSTM layers followed by a fully connected layer. Each LSTM layer contains 64 units with a dropout rate of 0.2 applied between them. The input sequences were padded to a uniform length of 100 time steps. The output of the second LSTM layer was fed into a fully connected layer with ReLU activation for classification. The model was trained using the Adam optimizer with a learning rate of 0.005. The categorical cross-entropy loss function was used for sequence classification. To prevent overfitting, early stopping was applied with a patience of 10 epochs on the validation loss. The batch size was set to 64, and the model was trained for a maximum of 100 epochs.

## 2. Hyperparameter Optimization

To identify optimal hyperparameters and achieve global convergence, we employed GridSearchCV and RandomizedSearchCV techniques from the sklearn.model_selection module. These methods systematically explore the hyperparameter space by performing a cross-validated search over a predefined set of parameter values. Specifically, a grid of hyperparameter values was defined for each model. The search space for the learning rate (lr) was [0.001, 0.01, 0.1], and for the number of hidden units (hidden_units), we considered [32, 64, 128]. The models were trained and validated using 5-fold cross-validation on the training dataset. The optimal hyperparameters were selected based on the highest average validation performance.

Additionally, to address the challenges of optimizing complex hyperparameters in deep learning models, three optimization algorithms were explored: 1) Ant Colony Optimization is a nature-inspired optimization algorithm that mimics the foraging behavior of ants. For ACO, we set the number of ants (n_ants) to 20 and the maximum number of iterations (n_iterations) to 100. The pheromone evaporation rate (pheromone_evaporation) was set to 0.1. The models were trained with the updated hyperparameters over each iteration; 2) Harris Hawk Optimization is a recent metaheuristic optimization algorithm that simulates the hunting behavior of hawks. For HHO, we initialized a population of 25 hawks. The exploration probability (exploration_prob) was set to 0.4, and the exploitation probability (exploitation_prob) was set to 0.6. The maximum number of iterations (n_iterations) was set to 50; 3) Bayesian Optimization is a probabilistic optimization technique that aims to find the optimal hyperparameters by balancing exploration and exploitation, thus minimizing the number of evaluations needed. For Bayesian Optimization, we initialized 10 random configurations and performed a total of 30 iterations. The acquisition function used was Expected Improvement (acq_func = "EI") with a kappa parameter of 1. The Gaussian Process kernel was set to Radial Basis Function (kernel = RBF).

The hyperparameter optimization processes were conducted using Python's scikit-learn library (version 0.24.1) for GridSearchCV and RandomizedSearchCV, and the scikit-optimize library for Bayesian Optimization. The deep learning models were implemented using the TensorFlow framework (version 2.12.0) with the Keras API (version 2.12.0). The optimal hyperparameters obtained from each optimization technique were then used to train the final models.

## 3. Feature engineering

Transcriptomic and proteomic profiles were selected as feature samples. To evaluate model generalization, we randomly split the samples into training and testing subsets, comprising 80% and 20% of the main dataset, respectively. To improve the predictive performance and interpretability of the model, we propose a feature engineering scheme called HDMPPK (HistGradientBoostingRegressor, DBSCAN, MinMaxScaler, principal component analysis, Particle Swarm Optimization, and K-Nearest Neighbors). HDMPPK approach is a multi-step feature engineering pipeline that involves the following components: 1) HistGradientBoostingRegressor Imputation: Missing values were addressed using the sklearn.ensemble.HistGradientBoostingRegressor to predict and impute the missing values accurately. 2）DBSCAN Anomaly Detection: Outliers were detected using the Density-Based Spatial Clustering of Applications with Noise (DBSCAN) algorithm. This step aimed to identify and handle potential anomalies in the dataset. 3) MinMaxScaler Normalization: Data normalization was performed using sklearn.preprocessing.MinMaxScaler to ensure that all features were on the same scale, preventing any feature from dominating the learning process. 4）PCA Feature Dimensionality Reduction: Principal Component Analysis (PCA) was employed to reduce feature dimensionality while retaining the most informative aspects of the data. 5）Particle Swarm Optimization (PSO) and K-Nearest Neighbors (KNN) Feature Selection: PSO was applied to optimize feature subsets for enhanced model performance. The KNN regressor was used to evaluate the fitness of feature subsets during the optimization process. In addition, SGL regression is also used for feature selection of the dataset to compare with the HDMPPK scheme. Feature datasets selected by the HDMPPK method were used to train SVR, KNR, CNN, and LSTM. The above algorithms are all implemented using the Scikit-learn library (version 0.24.1). The HDMPPK framework and the construction of various machine learning models are available at: <https://github.com/bixinyuJN/machine_learning>.

## 4. Reconstruction of *i*Bsu1209-ME model based on BsuMAC dataset predicted by ensemble model

The BB integrated model (gene transcription and protein translation is the input and cell growth rate is the output) is used to predict cell growth under different gene expression conditions in BsuMAC. Provides reliable cell growth rates for 496 gene expression profiles. The *i*Bsu1209-ME model was optimized based on the corresponding relationship between 496 gene expression and cell growth rate. Based on the experimental conditions reported in the literature as input, *i*Bsu1209-ME simulation predicts cell growth rate and gene expression under different conditions. Compare the expression of 496 genes and modify the code of the transcription module and translation module built by the *i*Bsu1209-ME model. Detailed information can be obtained from <https://github.com/bixinyuJN/BSUme>.

## 5. Modeling and simulation of *i*Bsu1209-ME

The *i*Bsu1209-ME model is built based on the COBRAme framework^[4]^, gene expression database of *Bacillus subtilis* (BSUme, constructed by this study) and et*i*Bsu1209.^[2]^ The *i*Bsu1209-ME building code is at https://github.com/bixinyuJN/BSUme. For the verification and simulation of the DLKcat model, we collected the enzymatic data (*k*cat) of *B. subtilis* in the BRENDA and SABIO-RK databases (Table S5). The DLKcat model simulated 650 proteins in *i*Bsu1209-ME, and the simulated values were compared to experimental data from above database, achieving a correlation of 0.86.^[1]^ Using the DLKcat model, we predicted orther unknown *k*cat across *i*Bsu1209-ME, expanding the enzymes dataset from 650 to 1847. To assess the simulation accuracy of *i*Bsu1209-ME metabolic flux and prediction capabilities, we input experimental values into the model and utilized pFBA and solveME algorithms to predict cell phenotypes and gene expression (LB medium, glucose+NH4^+^, malate+NH4^+^, fructose+NH4^+^, glycerol+NH4^+^, glucose+nitrate, glucose+glutamine, glucose+ornithine, glucose+adenine, glucose+malate+NH4^+^).^[3, 4]^ The simulation values were compared with the experimental values to obtain the prediction accuracy of the model.

## Supplementary References：

[1] F. Li, L. Yuan, H. Lu, G. Li, Y. Chen, M. K. Engqvist, E. J. Kerkhoven, J. Nielsen, *Nature Catalysis* 2022, **5**, 662.

[2] X. Bi, Y. Cheng, X. Xu, X. Lv, Y. Liu, J. Li, G. Du, J. Chen, R. Ledesma‐Amaro, L. Liu, *Biotechnology Bioengineering* 2023, **120**, 1623.

[3] B. M. Koo, G. Kritikos, J. D. Farelli, H. Todor, K. Tong, H. Kimsey, I. Wapinski, M. Galardini, A. Cabal, J. M. Peters, A. B. Hachmann, D. Z. Rudner, K. N. Allen, A. Typas, C. A. Gross, *Cell systems* 2017, **4**, 291.

[4] C. J. Lloyd, A. Ebrahim, L. Yang, Z. A. King, E. Catoiu, E. J. O’Brien, J. K. Liu, B. O. Palsson, PLoS computational biology 2018, **14**, e1006302.
